# Effects of Implementation of Infection Control Measures against COVID-19 on the Condition of Japanese Rural Nursing Homes

**DOI:** 10.3390/ijerph18115805

**Published:** 2021-05-28

**Authors:** Ryuichi Ohta, Yoshinori Ryu, Chiaki Sano

**Affiliations:** 1 Community Care, Unnan City Hospital, 96-1 Iida, Daito-cho, Unnan, Shimane 699-1221, Japan; yoshiyoshiryuryu.hpydys@gmail.com; 2 Department of Community Medicine Management, Faculty of Medicine, Shimane University, Izumo, Shimane 693-8501, Japan; sanochi@med.shimane-u.ac.jp

**Keywords:** COVID-19, emergency transportation, nursing home, day off, rural, Japan

## Abstract

This study aimed to clarify the effect of coronavirus disease (hereafter, COVID-19) control on patients’ health conditions and staff’s working conditions in rural nursing homes. An interventional study including all staff and patients in a rural nursing home was performed from 1 April 2019 to 31 March 2021. Infection control measures against COVID-19 were initiated on 1 April 2020. The primary outcome was the frequency of patients’ medical care visits to the outpatient and emergency departments. The secondary outcome was the number of days-off taken by staff. Each group (pre- and post-COVID-19 control groups) had 48 participants. The number of visits to the outpatient department reduced from the pre-COVID-19 to post-COVID-19 control period the difference in number of visits to the emergency department was not significant, due to the low statistical power. The number of days-off taken by the staff was increased from the pre-COVID-19 to post-COVID-19 control period. This is the first study investigating COVID-19 control measures in a rural nursing home. It may help reduce the number of patient visits to medical facilities without increasing the risk of emergencies. A strict health check of the staff can allow staff to take more days off in rural contexts.

## 1. Introduction

Nursing homes are one of the most vulnerable places during the coronavirus (COVID-19) pandemic. Nursing homes accommodate older patients who require exceptional care, and these patients with polypharmacy and multimorbidity are vulnerable to various infections [1,2]. Older patients with COVID-19 have been shown to have a high mortality, and there have been numerous casualties in this population during the pandemic [3,4]. Moreover, the incidence of COVID-19 in nursing homes has led to critical health situations in numerous older individuals, some of whom have succumbed to the infection [5,6]. Several waves of the COVID-19 pandemic have affected nursing homes in both developed and developing countries due to the difficulties in controlling interactions among people [7,8]. The initial stage of this pandemic has affected the lives of older individuals in particular, causing numerous deaths among them [7]. As evidence regarding COVID-19 prevention continues to accumulate, nursing homes have been strenuously focusing on infection control measures [9,10].

Intensive infection control measures in nursing homes can protect vulnerable older patients. The most important aspect is controlling contact among people [11,12]. Older patients have various types of diseases; therefore, treatment of their medical conditions requires regular monitoring from physicians and nurses [13]. As their routine care depends on the staff in nursing homes, the interaction between them is inevitable [14]. During such interactions, handwashing and wearing facemasks is mandatory, which can significantly reduce the transmission of infection [15]. Another spreader of COVID-19 has been visitors from outside the nursing homes. For strict infection control, their visits have had to be limited and based on strict guidelines [16,17]. Moreover, information and communication technology (ICT) has been critical in reducing contact between patients and their visitors and has been used in various settings [18]. Furthermore, the health conditions of both patients and staff in nursing homes should be monitored for symptoms such as fever and upper respiratory issues [19]. Staff who are symptomatic have been encouraged to take a day off and get tested for COVID-19, even if their symptoms are not severe and they are able to work in routine situations [20]. Specifically, multimodal approaches are required for the prevention of COVID-19 spread in nursing homes.

Intensive infection control can affect the conditions of nursing homes, especially in rural settings with scant healthcare resources [21,22]. Infection control requires limiting medical care in medical facilities and reducing the duration of care provided to patients, which can lead to low-quality medical care and the risk of missing signs of acute conditions in patients [23,24,25]. Additionally, as few healthcare resources are available in nursing homes in rural areas, the staff cannot take a day off if they are symptomatic [26]. Thus, a clarification of the patients’ health conditions and the staff’s working conditions in rural areas can lead to a better understanding of strict COVID-19 control in rural nursing homes, which can contribute to the appropriate management of rural nursing homes during the pandemic. However, the scope of this practice has not been sufficiently investigated. Therefore, the purpose of this study was to identify the effect of COVID-19 control on the patients’ health conditions and the staff’s working conditions in rural nursing homes.

## 2. Materials and Methods

### 2.1. Participants

This interventional study involving all the staff and patients in a rural nursing home was performed from 1 April 2019 to 31 March 2021. As an intervention, infection control measures against COVID-19 were initiated on 1 April 2020. The study participants were patients and staff living or working in a rural nursing home; the pre-COVID-19 and post-COVID-19 control groups were defined as staff and patients living or working therein before and after the implementation of infection control measures, respectively. The pre-COVID-19 control group was further defined as patients and staff who lived or worked in the nursing home between 1 April 2019 and 31 March 2020.

### 2.2. Setting

The study was conducted at Kakeya Clinic (a rural clinic) and Egaonosato Nursing Home. Kakeya Clinic is located in Kakeya Town, Unnan, Shimane prefecture, which is situated in the westernmost part of Unnan City, 30 km away from Unnan City Hospital, which is the only general hospital in the city. There are three registered family physicians and three nurses at the clinic. The family physicians work at both Unnan City Hospital and the clinic, where they visit once or twice a week. The clinic does not have beds for admission, and emergency cases are transferred to Unnan City Hospital. Egaonosato Nursing Home is located near the clinic and can accommodate 40 dependent patients. The nursing home has 4 nurses, 32 care workers, and 16 clerks. The clinic physicians are charged with providing medical care to the nursing home patients. Once a week, the physicians visit the nursing home and examine the patients. The nursing home nurses can call the clinic whenever the patients have emergency medical symptoms [27]. During the study period, 287 persons were infected with COVID-19 in Shimane prefecture.

### 2.3. Application of Infection Control for the Prevention of COVID-19

From 1 April 2020 onward, infection control measures for the prevention of COVID-19 were initiated in the nursing home. Based on discussions among the staff at the nursing home, the clinic physicians, and an advanced registered nurse for infection at Unnan City Hospital, three specific measures were implemented: contact limitation, daily monitoring of the staff’s health conditions, and ICT usage.

### 2.4. Contact Limitation

To reduce the risk of infection transmission, care workers wore facemasks, plastic gloves, and face shields, and used hand sanitizers every time they cared for their patients. The frequency of care was reduced from three times/day to two times/day. Regarding mealtimes, the patients usually ate their food in the lounges; however, they were now required to eat in their respective rooms during the pandemic. Further, the patients’ families were restricted from meeting the patients, except in emergency situations.

### 2.5. Daily Monitoring of the Staff’s Health Conditions

The staff and clinic physicians were required to monitor their fever and symptoms daily and note down their conditions on a checklist. The checklists were monitored, and if they had mild symptoms or fever >37 °C, they were not permitted to work in the nursing home.

### 2.6. Usage of ICT

To share patient information between the clinic and nursing home, an ICT system called “Mame-net” was used, which was established by the local government of the Shimane prefecture in Japan. Using this system, the clinic and nursing home can share information regarding the patients’ medical and care conditions, including acute and chronic changes in the patients’ medical conditions. After posting the patients’ information via the ICT system, a computer-generated notification mail is automatically sent to all medical and care professionals involved in the patient’s care. The physicians, nurses, and care workers share patient information predominantly via this system. If any patient shows emergency symptoms, the nurses are required to call the physicians directly by phone, not via the ICT system [27]. The previous study shows the mitigation of the anxiety of nurses and care workers in nursing homes [27]. In this ongoing pandemic, there is an increase in pressure for nurses and care workers to identify symptoms in their patients. For the mitigation of this pressure, the ICT system was usable in any situation. The patients’ families can interact with the patients via the ICT system, and the staff respond to the families’ questions regarding their patients through this system as well.

### 2.7. Data Collection

The patients’ background information was obtained from the electronic medical records of the clinic. The background information included age, sex, serum albumin level, renal function, level of dependent care based on the Japanese long-term insurance system (Stages 1–5; 1: least dependent and 5: completely dependent), medical histories, the Charlson comorbidity index (CCI) calculated from the medical histories [28], number of medicines, and history of previous admission to hospitals within the last 6 months. The primary outcome was the patients’ frequency of medical care visits to the outpatient and emergency departments. The patients’ medical care visits to the outpatient and emergency departments during each month was calculated. The secondary outcome was the number of days-off taken by the staff. The number of days-off taken by the staff in each month was calculated.

### 2.8. Statistical Analysis

The differences in participant characteristics, frequency of patients’ medical care visits to the outpatient and emergency departments, and the number of days-off taken by the staff between the pre- and post-COVID-19 control groups were analyzed using t-tests and chi-squared tests. CCI was categorized binomially (≤5 or >5) to assess the severity of medical conditions [28]. For all comparisons, statistical significance was set at *p* < 0.05. From the effect size estimation, a minimum of 40 participants were required in each group, using α (alpha) = 0.05, β (beta) = 0.10 (power of 90%), and a between-group difference of 20% in the number of patients’ medical care visits to the outpatient and emergency departments. Cases with missing data were excluded from the analysis. All statistical analyses were performed using EZR (Saitama Medical Center, Jichi Medical University, Saitama, Japan), which is a graphical user interface for R (The R Foundation, Vienna, Austria) [29].

### 2.9. Ethics Approval

The participants were informed that the data collected in this study would only be used for research purposes. Participants were also informed about the aims of this study, how the data would be disclosed, and that their personal information would be protected, following which, they provided written informed consent. This study was approved by the Rural City Hospital Clinical Ethics Committee (approval number: 2021004).

## 3. Results

### 3.1. Demographic Data

The total number of participants was 96 (48 in each group–the pre- and post-COVID-19 control groups). The average age in the pre- and post-COVID-19 groups was 88.92 (standard deviation [SD] = 7.24) and 90.38 (SD = 6.08) years, respectively. There was no difference in the participants’ background information between the two groups (Table 1). There was no difference in the CCI score between the two groups. Throughout the study period, no case was diagnosed with COVID-19.

### 3.2. Patients’ Medical Care Visits to the Outpatient and Emergency Departments

The total number of medical care visits to the outpatient department reduced from the pre-COVID-19 to post-COVID-19 control period (261 vs. 210) (Table 2); however, the difference was not statistically significant (*p* = 0.111) (Table 3). Further, there was no significant difference in medical care visits to the emergency department between the two periods (66 vs. 62) (Table 2) (*p* = 0.761; Table 3).

### 3.3. Number of Days-Off Taken by the Staff

The total number of days-off taken by the staff increased from the pre-COVID-19 to post-COVID-19 control period (79 vs. 155) (Table 4). However, the difference was not statistically significant (*p* = 0.269) (Table 3).

## 4. Discussion

This study showed that the implementation of strict infection control measures against COVID-19 in a rural nursing home can reduce the contact between the nursing home patients and healthcare staff, without an increase in the number of patients experiencing emergency situations. Additionally, the staff working in the rural nursing home took more days off during the pre-COVID-19 control period, which implies that the COVID-19 pandemic might make people more sensitive to their symptoms and encourage them to take leave from work. Appropriate implementation of infection control measures against COVID-19 may reduce the effective care provided to nursing home patients and optimize the working conditions of the staff in rural nursing homes.

Infection control measures against COVID-19 can be implemented in rural nursing homes without major complications. In this study, the implementation reduced the number of visits to the outpatient department without increasing the visits to the emergency department. Nursing home patients typically have various diseases and are vulnerable to acute conditions. Nursing homes have been significantly affected by the COVID-19 pandemic, and there have been numerous casualties despite strict infection control measures and a sufficient workforce [30,31]. Strict infection control measures against the pandemic have been shown to be effective in preventing the spread of COVID-19 in nursing homes; however, the contact limitations between the medical staff and the patients’ families can induce more stress in the patients, causing potential emergency situations [32,33]. In this study, our implementation of infection control included the use of ICT, with constant communication among the medical staff that allowed them to monitor subtle changes in the patients’ conditions and control them in the early stages [34]. A previous study showed that increasing the prevalence of ICT usage in nursing homes can reduce the number of emergency transportation requirements and number of COVID-19 cases [34,35]. Due to the low statistical power of this study, we did not observe any statistically significant results. Moreover, rural healthcare workers and families might face difficulties in using ICT [36]. As ICT-driven infection control measures against COVID-19 can be effective in monitoring nursing home patients, future studies should investigate this issue and ICT education in larger samples.

Controlling the staff’s working conditions in rural nursing homes is critical, and our study’s findings may be useful in improving the health conditions of nursing home medical staff. In our study, the rural nursing home staff took more days off during the post-COVID-19 control period than during the pre-COVID-19 control periods by controlling their working conditions. A previous study showed that healthcare professionals in nursing homes easily got exhausted from the COVID-19 control measures [37,38]. This exhaustion of healthcare professionals may lead to physical and mental damage, resulting in a poor quality of care [26,39]. Healthcare workers such as care providers, nurses, and physicians have to work continuously during this pandemic, with a great exposure to COVID-19 [23,40]. Strict infection control measures can decrease the morbidity and mortality of healthcare workers [41]. Their health conditions should be checked intensively, and they should be given opportunities for rest. However, due to the lack of healthcare resources in rural settings, healthcare workers have to work hard to compensate for this lack. In this study, by controlling the amount of work through limitation of the contact times with patients and other workers, it was easier for healthcare workers with mild symptoms to take days off, compared with the previous situation. The adjustment of working conditions can be a challenge for organizations; however, the COVID-19 pandemic should urge healthcare institutions to change their working styles and systems. Future studies should investigate the process of changing working systems and culture, and the effectiveness of the styles and changes on the health conditions of patients and health workers.

This study has several limitations. Since it was performed in a single nursing home located in a Japanese rural area, the study setting cannot be considered representative of rural medicine in developing and developed countries, in terms of the lack of medical resources, aging societies, and isolation of older people. Future studies should investigate these constructs in other rural settings, such as on remote islands or in developing countries. Another limitation pertains to the sampling method. Potential confounding factors were included in this study; however, randomization of the sampling process could further address the potential confounding factors. Future studies should implement randomization to overcome this limitation.

## 5. Conclusions

COVID-19 control may reduce the requirement for patients in rural nursing homes to visit medical facilities, without increasing their risk of emergency situations. A strict health check of the staff as an infection control measure can promote effective working, allow staff to take more days off, and more focus on patients’ health conditions without the anxiety of healthcare staff in rural nursing homes. Continuous implementation of the infection control measures, based on the conditions of COVID-19 pandemic, can mitigate infection risks of patients and health care workers in rural nursing homes and improve working conditions of healthcare workers in rural nursing homes through effective collaboration with medical staff of rural medical institutions.

## Figures and Tables

**Table 1:** Demographic data of the patients.

	Group		
Factor	Pre-COVID Control (2019−2020)	Post-COVID Control (2020−2021)	*p* Value
Age, average (SD)	88.92 (7.24)	90.38 (6.08)	0.288
Sex (%)	11 (22.9)	11 (22.9)	1
Albumin, average (SD)	3.46 (0.40)	3.50 (0.40)	0.559
eGFR, average (SD)	77.11 (29.57)	72.40 (30.54)	0.445
Medicines, average (SD)	5.79 (2.49)	6.31 (2.75)	0.334
Care level (%)			
4	9 (18.8)	8 (16.7)	1
5	39 (81.2)	40 (83.3)	
Previous admission (%)	21 (43.8)	29 (60.4)	0.152
CCI	6.42 (1.40)	6.31 (1.19)	0.638
Asthma (%)	2 (4.2)	1 (2.1)	1
Brain hemorrhage (%)	5 (10.4)	4 (8.3)	1
Brain infarction (%)	13 (27.1)	12 (25.0)	1
Cancer (%)	3 (6.2)	2 (4.2)	1
Connective tissue disease (%)	0 (0.0)	1 (2.1)	1
COPD (%)	1 (2.1)	1 (2.1)	1
Dementia (%)	47 (97.9)	47 (97.9)	1
DM (%)	10 (20.8)	7 (14.6)	0.594
Heart failure (%)	13 (27.1)	12 (25.0)	1
Hemiplegia (%)	2 (4.2)	2 (4.2)	1
Kidney diseases (%)	8 (16.7)	11 (22.9)	0.609
Liver diseases (%)	0 (0.0)	1 (2.1)	1
Myocardial infarction (%)	7 (14.6)	6 (12.5)	1
Peptic ulcer (%)	1 (2.1)	1 (2.1)	1

eGFR, estimated glomerular filtration rate; CCI, Charlson’s Comorbidity Index; COPD, chronic obstructive pulmonary disease; DM, diabetes mellitus; SD, standard deviation.

**Table 2:** Annual medical care visits to the outpatient department during the pre- and post-COVID period.

	Pre-COVID (2019–2020)				Post-COVID (2020–2021)			
	OD	ED	OD/Patient	ED/Patient	OD	ED	OD/Patient	ED/Patient
April	23	3	0.479	0.063	16	4	0.333	0.083
mMay	32	4	0.667	0.083	9	3	0.188	0.063
June	17	6	0.354	0.125	16	4	0.333	0.083
July	19	7	0.396	0.146	10	9	0.208	0.188
August	25	3	0.521	0.063	14	4	0.292	0.083
September	26	4	0.542	0.083	28	8	0.583	0.167
October	19	5	0.396	0.104	19	5	0.396	0.104
November	14	12	0.292	0.250	22	2	0.458	0.042
December	27	8	0.563	0.167	30	6	0.625	0.125
January	17	5	0.354	0.104	19	4	0.396	0.083
February	14	7	0.292	0.146	11	3	0.229	0.063
March	28	2	0.583	0.042	16	10	0.333	0.208
Total	261	66	5.438	1.375	210	62	4.375	1.29

OD, outpatient department; ED, emergency department.

**Table 3:** Difference between the pre- and post-COVID 19 periods.

	Pre-COVID (2019–2020)	Post-COVID (2020–2021)	*p*-Value
Outpatient department			
monthly, average (SD)	21.75 (5.89)	17.5 (6.61)	
per patient, monthly, average (SD)	0.45 (0.12)	0.37 (0.14)	0.111
Emergency department			
monthly, average (SD)	5.50 (2.75)	5.18 (2.55)	
per patient, monthly, average (SD)	0.115 (0.06)	0.108 (0.05)	0.761
Staff day off			
monthly, average (SD)	6.58 (10.65)	12.92 (16.17)	
per staff, monthly, average (SD)	0.13 (0.20)	0.25 (0.31)	0.269

**Table 4:** Annual days-off taken by the staff during the pre-COVID-19 and post-COVID-19 period.

	Pre-COVID (2019–2020)		Post-COVID (2020–2021)	
	Days Off	Days Off/Staff	Days Off	Days Off/Staff
April	0	0.00	0	0.00
May	0	0.00	0	0.00
June	15	0.29	0	0.00
July	34	0.65	0	0.00
August	4	0.08	26	0.50
September	9	0.17	30	0.58
October	0	0.00	26	0.50
November	0	0.00	3	0.06
December	17	0.33	0	0.00
January	0	0.00	3	0.06
February	0	0.00	20	0.38
March	0	0.00	47	0.90
Total	79	1.52	155	2.98

## Data Availability

The datasets used and/or analyzed during the current study may be obtained from the corresponding author upon reasonable request.
